# Measures to strengthen primary health-care systems in low- and middle-income countries

**DOI:** 10.2471/BLT.20.252742

**Published:** 2020-09-28

**Authors:** Etienne V Langlois, Andrew McKenzie, Helen Schneider, Jeffrey W Mecaskey

**Affiliations:** a Partnership for Maternal, Newborn and Child Health, World Health Organization, Avenue Appia 20, 1211 Geneva 27, Switzerland.; b DAI Global Health, London, England.; c South African Medical Research Council Health Services to Systems Unit, University of the Western Cape School of Public Health, Cape Town, South Africa.

## Abstract

Primary health care offers a cost–effective route to achieving universal health coverage (UHC). However, primary health-care systems are weak in many low- and middle-income countries and often fail to provide comprehensive, people-centred, integrated care. We analysed the primary health-care systems in 20 low- and middle-income countries using a semi-grounded approach. Options for strengthening primary health-care systems were identified by thematic content analysis. We found that: (i) despite the growing burden of noncommunicable disease, many low- and middle-income countries lacked funds for preventive services; (ii) community health workers were often under-resourced, poorly supported and lacked training; (iii) out-of-pocket expenditure exceeded 40% of total health expenditure in half the countries studied, which affected equity; and (iv) health insurance schemes were hampered by the fragmentation of public and private systems, underfunding, corruption and poor engagement of informal workers. In 14 countries, the private sector was largely unregulated. Moreover, community engagement in primary health care was weak in countries where services were largely privatized. In some countries, decentralization led to the fragmentation of primary health care. Performance improved when financial incentives were linked to regulation and quality improvement, and community involvement was strong. Policy-making should be supported by adequate resources for primary health-care implementation and government spending on primary health care should be increased by at least 1% of gross domestic product. Devising equity-enhancing financing schemes and improving the accountability of primary health-care management is also needed. Support from primary health-care systems is critical for progress towards UHC in the decade to 2030.

## Résumé

Les soins de santé primaires offrent l'itinéraire le plus économique vers une couverture maladie universelle (CMU). Pourtant, les systèmes dont ils dépendent demeurent fragiles dans de nombreux pays à faible et moyen revenu. La plupart du temps, ils sont incapables de fournir un modèle de soins intégral, intégré et centré sur la personne. Nous avons donc analysé les systèmes de soins de santé primaires dans 20 pays à faible et moyen revenu en adoptant une approche partiellement basée sur la réalité. Les options de renforcement des systèmes de soins de santé primaires ont été identifiées par le biais d'une analyse de contenu thématique. Nous avons constaté que: (i) malgré le fardeau de plus en plus lourd des maladies non transmissibles, nombre de pays à faible et moyen revenu ne possédaient pas les fonds suffisants pour assurer des services de prévention; (ii) les professionnels de santé au sein des communautés manquaient fréquemment de ressources, de soutien et de formation; (iii) les frais non remboursables dépassaient 40% des dépenses de santé dans la moitié des pays étudiés, ce qui entraîne des inégalités; et enfin, (iv) les régimes d'assurance maladie étaient entravés par la fragmentation des systèmes publics et privés, le sous-financement, la corruption et la piètre mobilisation des travailleurs informels. Dans 14 pays, le secteur privé n'était pratiquement soumis à aucune réglementation. Par ailleurs, l'engagement communautaire dans les soins de santé primaires était dérisoire dans les États où les services étaient majoritairement privatisés. Dans certains pays, la décentralisation avait débouché sur une fragmentation des soins de santé primaires. Les performances se révélaient meilleures lorsque des avantages financiers avaient trait à la réglementation et à l'amélioration de la qualité, et l'implication était forte au sein de la communauté. Le processus d'élaboration des politiques devrait être accompagné des ressources nécessaires pour l'instauration d'un système de soins de santé primaires, et les gouvernements devraient accroître leurs dépenses en la matière d'au moins 1% du produit intérieur brut. Il est également impératif de définir des régimes de financement favorisant l'équité et de promouvoir la fiabilité de la gestion des soins de santé primaires. La contribution des systèmes de soins de santé primaires est essentielle à la progression vers une CMU à l'horizon 2030.

## Resumen

La atención primaria de salud brinda una vía rentable para lograr la cobertura sanitaria universal (CSU). Sin embargo, los sistemas de atención primaria de salud son deficientes en muchos países de ingresos medios y bajos y con frecuencia no ofrecen una atención integral y centrada en las personas. Se analizaron los sistemas de atención primaria de salud en 20 países de ingresos medios y bajos mediante un enfoque semifundamentado. Se determinaron las alternativas para fortalecer los sistemas de atención primaria de salud por medio de un análisis de contenido temático. Se concluyó que: i) a pesar de la creciente carga de las enfermedades no transmisibles, muchos países de ingresos medios y bajos no disponían de fondos para los servicios preventivos; ii) con frecuencia los profesionales sanitarios de la comunidad carecían de recursos, de apoyo y de capacitación; iii) los gastos directos superaban el 40 % del gasto total en salud en la mitad de los países analizados, lo que afectaba a la equidad; y iv) los planes de seguro médico presentaban dificultades debido a la fragmentación de los sistemas públicos y privados, la falta de financiamiento, la corrupción y la escasa participación de los trabajadores informales. La mayor parte del sector privado de 14 países no estaba regulado. Además, la participación de la comunidad en la atención primaria de salud era muy reducida en los países donde los servicios estaban privatizados en gran medida. Por otra parte, la descentralización de la atención primaria de salud causó la fragmentación de la misma en algunos países. La rentabilidad mejoró cuando los incentivos financieros se vincularon con la regulación y el mejoramiento de la calidad, además de que la participación de la comunidad fue significativa. La formulación de las políticas debería contar con el apoyo de recursos suficientes para prestar los servicios de atención primaria de salud y el gasto público en atención primaria de salud debería aumentar por lo menos en un 1 % del producto interno bruto. Asimismo, es necesario elaborar planes de financiamiento que aumenten la equidad y mejoren la rendición de cuentas de la gestión de la atención primaria de salud. El apoyo de los sistemas de atención primaria de salud es fundamental para avanzar hacia la CSU de aquí a 2030.

## ملخص

تقدم الرعاية الصحية الأولية وسيلة فعالة من حيث التكلفة لتحقيق تغطية صحية شاملة (UHC). إلا أن أنظمة الرعاية الصحية الأولية ضعيفة في العديد من البلدان منخفضة الدخل ومتوسطة الدخل، وغالبًا ما تفشل في توفير رعاية شاملة ومتكاملة ومرتكزة على الإنسان. قمنا بتحليل أنظمة الرعاية الصحية الأولية في 20 بلدًا منخفض الدخل ومتوسط الدخل باستخدام أسلوب شبه أساسي. تم تحديد خيارات لتعزيز أنظمة الرعاية الصحية الأولية من خلال تحليل المحتوى المواضيعي. توصلنا إلى ما يلي: (1) على الرغم من العبء المتزايد للأمراض غير المعدية، فإن العديد من البلدان منخفضة الدخل ومتوسطة الدخل افتقرت إلى أموال الخدمات الوقائية؛ و(2) كان العاملون في القطاع الصحي المجتمعي في كثير من الأحيان يعانون من نقص الموارد وضعف الدعم والافتقار إلى التدريب؛ و(3) تجاوز الإنفاق الشخصي 40% من إجمالي الإنفاق الصحي في نصف البلدان التي شملتها الدراسة، وهو ما أثر على العدالة؛ و(4) تعرضت أنظمة التأمين الصحي بسبب تجزئة الأنظمة العامة والخاصة، ونقص التمويل، والفساد، وضعف مشاركة العمال غير الرسميين. كان القطاع الخاص غير منظم بشكل كبير في 14 بلدًا. بالإضافة إلى ذلك، كانت مشاركة المجتمع في الرعاية الصحية الأولية ضعيفة في البلدان التي كانت الخدمات مخصخصة فيها إلى حد كبير. أدت اللامركزية في بعض البلدان إلى تجزئة الرعاية الصحية الأولية. تحسن الأداء عندما تم ربط الحوافز المالية بالتنظيم وتحسين الجودة، وكانت مشاركة المجتمع قوية. يجب دعم وضع السياسات بواسطة الموارد الكافية لتنفيذ الرعاية الصحية الأولية، كما يجب زيادة الإنفاق الحكومي على الرعاية الصحية الأولية بنسبة 1% على الأقل من الناتج المحلي الإجمالي. كما أن هناك حاجة إلى وضع مخططات تمويل لتعزيز العدالة، وتحسين المساءلة بخصوص إدارة الرعاية الصحية الأولية. يعد الدعم الناتج من أنظمة الرعاية الصحية الأولية أمرًا حيويًا لإحراز التقدم نحو تحقيق التغطية الصحية الشاملة (UHC) في العقد الحالي حتى عام 2030.

## 摘要

初级卫生保健为实现全民健康覆盖 (UHC) 提供了一种经济有效的途径。然而在许多中低收入国家，初级卫生保健体系非常薄弱，常常无法提供全面的以人为本的综合保健。我们利用半扎根的研究方法分析了 20 个中低收入国家的初级卫生保健体系。通过分析主题内容确定了加强初级保健体系的备选方案。我们发现：(I) 尽管非传染性疾病负担日益沉重，但许多中低收入国家仍缺乏预防服务资金；(ii) 社区卫生工作者通常资源配备不足，无法获得良好的支持，并且缺乏相应培训；(iii) 在所研究的一半国家中，医疗自费超出卫生支出总额的 40％，不利于实现公平；(iv) 公共和私人体系分散、资金不足、腐败以及非正式员工参与不足，阻碍了健康保险计划的实施。14 个国家中，私营部门基本上不受监管。此外，服务基本私有化的国家，社区很少参与初级卫生保健。一些国家权力下放，促使其初级保健分散。当财政激励措施与法规和质量提升相结合，，并且社区参与度增强时，绩效便会有所改善。应该为初级卫生保健的决策制定提供充足的资源，并将政府在初级卫生保健方面的支出至少增加国内生产总值的 1％。还需要制定提升公平的融资方案，加强初级卫生保健管理的问责。初级卫生保健体系的支持对于在截止 2030 年的十年中实现 UHC 至关重要。

## Резюме

Первичная медико-санитарная помощь предлагает экономически эффективный метод обеспечения всеобщего охвата услугами здравоохранения (ВОУЗ). Тем не менее системы первичной медико-санитарной помощи слабо развиты во многих странах с низким и средним уровнем доходов и часто не способны обеспечить комплексное, ориентированное на людей медицинское обслуживание. Авторы проанализировали системы первичной медико-санитарной помощи в 20 странах с низким и средним уровнем доходов, используя метод полуобоснованной теории. Варианты укрепления систем первичной медико-санитарной помощи были определены в ходе анализа тематического содержания. Авторы выяснили следующее: (i) несмотря на растущее бремя неинфекционных заболеваний, многие страны с низким и средним уровнем доходов испытывают недостаток средств для профилактических услуг; (ii) работники здравоохранения на уровне сообществ часто не имеют достаточного количества ресурсов, поддержки и квалификации; (iii) расходы из собственных средств превышают 40% от общих расходов в сфере здравоохранения в половине исследованных стран, что способствует увеличению неравенства; (iv) работе схем медицинского страхования препятствует раздробленность государственных и частных систем, недостаток финансирования, коррупция и низкая степень участия неофициальных работников. В 14 странах частный сектор практически не регулировался. Более того, участие населения в первичной медико-санитарной помощи было низким в странах со значительной степенью приватизации услуг. В некоторых странах децентрализация привела к раздробленности сферы первичной медико-санитарной помощи. Показатели улучшились, когда финансовое стимулирование было связано с регулированием и повышением качества услуг, а также активным участием местного населения. Формирование политики должно подкрепляться соответствующими ресурсами для реализации первичной медико-санитарной помощи, а государственные расходы на первичную медико-санитарную помощь должны быть увеличены как минимум на 1% от валового внутреннего продукта. Также необходимо разработать схемы финансирования, способствующие уменьшению неравенства, и повысить подотчетность в управлении первичной медико-санитарной помощью. Поддержка со стороны систем первичной медико-санитарной помощи имеет решающее значение для достижения ВОУЗ в период до 2030 года.

## Introduction

Health systems around the world are facing increasingly complex challenges, such as the growing burden of chronic noncommunicable disease and related commercial determinants of health (e.g. the marketing of tobacco and unhealthy food), new epidemics and antimicrobial resistance. As a result, the focus has shifted from curative care to health promotion and disease prevention, and new models of primary health-care service delivery, financing and governance have been developed. 

Primary health-care systems are fundamental for responding to pandemics, such as the coronavirus disease 2019 (COVID-19) pandemic, and for maintaining essential health services. Moreover, primary health-care policies and interventions can enhance equity and support disadvantaged groups that have little coverage by essential services.^1^^,^^2^ Strengthening primary health care has been shown to improve population health outcomes and reduce all-cause mortality and is a cost-effective strategy for achieving universal health coverage (UHC).^2^^–^^6^ Primary health care is uniquely placed to provide the spectrum of care required to meet the majority of a population’s health needs, to provide services for communities locally and to address evolving needs.^6^ This adaptive ability contributes to the responsiveness and resilience of health systems, particularly in times of crisis.

In response to the 2018 Declaration of Astana,^7^ there has been a push for a renewed commitment to primary health care globally. There is an increasing recognition that achieving health-related sustainable development goals, including UHC, will not be possible without stronger primary health-care systems. In 2019, the World Health Assembly adopted a resolution that recognized the role of primary health care in providing health services throughout a person’s life course, including prevention, treatment, rehabilitation and palliative care.^8^ However, primary health-care systems are weak in many low- and middle-income countries and fail to provide high-quality, comprehensive, people-centred, integrated care. Often, systems are compromised by under-resourcing, fragmentation and poor governance. The priority assigned to primary health care and the resources provided vary greatly between countries: during 2011 to 2018, the proportion of annual government spending dedicated to primary health care by low- and middle-income countries ranged from 2 to 56%.^9^ Health services for poor and marginalized groups are often badly coordinated and inconsistent,^10^ and development aid and vertical programming (which focuses on disease-specific interventions) frequently increase the fragmentation of primary health care.^11^ Key governance challenges in primary health care include: poor regulation linked to low care quality, and a lack of transparency, efficiency and accountability in resource allocation and use.^12^

Traditionally, the core primary health-care principles of community engagement and empowerment, and of strengthening local health systems have been neglected at the expense of innovative and rapid interventions.^13^ To address this imbalance, the Declaration of Astana called for action to strengthen the three pillars of primary health care: (i) primary care and essential public health functions as the core of integrated health services; (ii) empowered people and communities; and (iii) multisectoral policies and actions.^7^

In low- and middle-income countries, the policies and system reforms needed to develop a more comprehensive and responsive primary health-care system must be supported by context-appropriate evidence. At present, there are gaps in knowledge about key aspects of primary health-care systems, including: quality, safety and performance management; policies and governance; organization and models of care; and financing.^14^ To address these gaps, the Alliance for Health Policy and Systems Research – an international partnership hosted by the World Health Organization (WHO) – developed an initiative called Primary Health Care Systems (PRIMASYS).^15^ The purpose of the initiative was to advance the science of primary health care in low- and middle-income countries and thereby support efforts to strengthen primary health-care systems and improve the implementation, effectiveness and efficiency of primary health-care interventions. To that end, PRIMASYS spearheaded the production of case studies of primary health-care policy and systems in 20 low- and middle-income countries.

Our aim was to use data from these 20 case studies to conduct a multicountry analysis of the system-level determinants of primary health-care performance and to derive lessons for primary health-care implementation, policy-making and systems reform that can aid efforts to strengthen primary health care in low- and middle-income countries.

## Development of case studies

In 2015, the Alliance for Health Policy and Systems Research convened an expert consultation on primary health-care systems in low- and middle-income countries, which involved global and country experts, including policy-makers and researchers from low- and middle-income countries.^16^ The PRIMASYS conceptual framework was adopted to guide the development and reporting of primary health-care system case studies.^17^ Between 2016 and 2018, studies were produced for 20 selected countries: Bangladesh, Cameroon, Colombia, Ethiopia, Georgia, Ghana, Indonesia, Kenya, Lebanon, Mexico, Mongolia, Nigeria, Pakistan, Peru, Rwanda, South Africa, Sri Lanka, Thailand, Uganda and the United Republic of Tanzania.^15^ Of the 20, four (20%) were low-income countries, nine (45%) were lower-middle-income countries and seven (35%) were upper-middle-income countries. The main population and primary health-care system characteristics for each of the 20 PRIMASYS countries are listed in Table 1.

**Table 1:** Country characteristics, case studies of primary health-care systems in 20 low- and middle-income countries, 2014–2018

Country	Population in 2018,^18^ no. in millions	Health expenditure in 2014,^19^ % of GDP	Nursing and midwifery personnel in 2017,^a,^^20^ no. per 10 000 population
**Low-income countries**			
Ethiopia	109.2	4.9	8.4
Rwanda	12.3	7.5	8.3^b^
Uganda	42.7	7.2	6.3^b^
United Republic of Tanzania	56.3	5.6	4.1^c^
**Lower-middle-income countries**			
Bangladesh	161.4	2.8	3.1
Cameroon	25.2	4.1	9.3^d^
Georgia	3.7	7.4	40.9^b^
Ghana	29.8	3.6	12.0
Indonesia	267.7	2.9	20.6
Kenya	51.4	5.7	15.4^c^
Mongolia	3.2	4.7	39.8
Nigeria	195.9	3.7	14.5^e^
Pakistan	212.2	2.6	5.0^b^
**Upper-middle-income countries**			
Colombia	49.7	7.2	12.6
Lebanon	6.3	6.4	26.4
Mexico	126.2	6.3	29.0^f^
Peru	32.0	5.5	13.5^f^
South Africa	57.8	8.8	35.2
Sri Lanka	21.7	3.5	21.2^f^
Thailand	69.4	4.1	29.6

GDP: gross domestic product.

^a^ Figures are for 2017 unless otherwise noted.

^b^ 2015.

^c^ 2014.

^d^ 2011.

^e^ 2013.

^f^ 2016.

The case studies were developed using a mixed-methods approach, which involved secondary data sets and primary data collected through engagement with primary health-care stakeholders, including in-depth interviews and focus group discussions. When primary health-care-specific indicators were not available as secondary data, the country teams produced estimates using the latest available validated metrics. Studies were reported using a standardized template.^21^

## Multicountry analysis

We carried out the data analysis using a semi-grounded approach and applied two primary health-care frameworks as coding matrices in the deductive component: the PRIMASYS framework and the Primary Health Care Performance Initiative framework.^22^ The domains analysed using these frameworks are presented in Box 1. Data on the frameworks’ domains were further analysed using an inductive process that allowed themes to emerge. Thematic content analysis of the 20 case studies was conducted using ATLAS.ti (ATLAS.ti Scientific Software Development GmbH, Berlin, Germany). Two investigators (AM and JM) performed the coding for case studies using codes from the two frameworks. Once coded, each domain was subcategorized using an inductive process to further interrogate the data. In addition, data were synthesized to identify key insights for strengthening and transforming primary health-care systems. Where appropriate, we augmented the analysis with data from WHO’s Global Health Observatory, including data on health financing and human resources for health. Ethics approval was obtained from WHO’s Ethics Review Committee (ERC.0003121).

Box 1 **Domains used of the PRIMASYS framework and the Primary Health Care Performance Initiative framework**

The PRIMASYS framework included: (i) primary health-care policy and systems domains (e.g. primary health-care system reform successes and failures); (ii) system governance and architecture; (iii) financing; (iv) human resources; (v) planning, implementation, monitoring, evaluation and information systems; (vi) regulatory processes; (vii) community participation; (viii) service delivery; (ix) infrastructure and supplies; and (x) primary health-care system strengthening priorities.

The Primary Health Care Performance Initiative framework was used to assess data on the domains of: (i) primary health-care systems (i.e. governance and leadership, health financing and adjustment to population health needs); (ii) inputs, such as drugs, supplies, infrastructure, health management information systems, workforce and funds; and (iii) service delivery, including access to care, care availability, people-centred care, organization and management.

PRIMASYS: Primary Health Care Systems.

### Disease burden

**Fig. 1:**
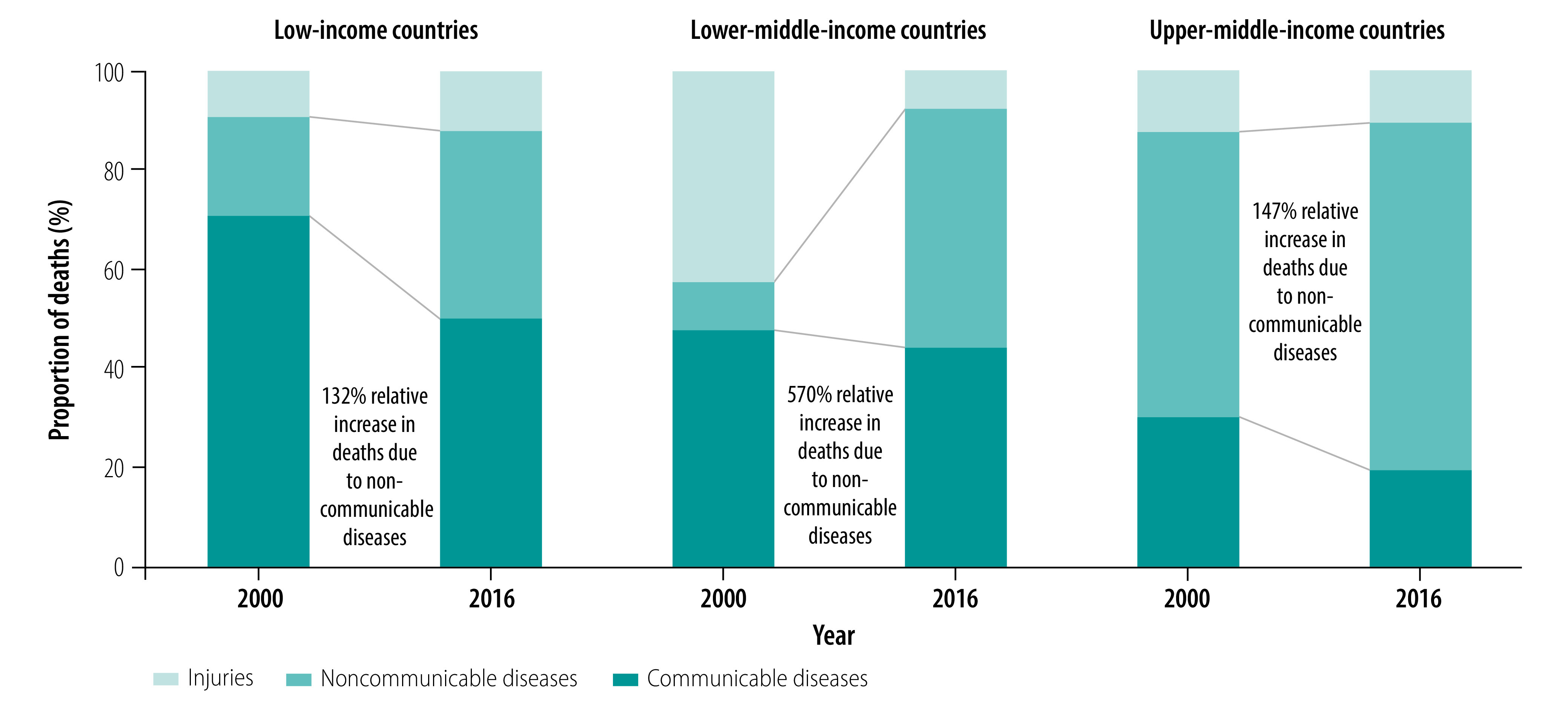
Deaths in low- and middle-income countries, by cause, 2000 and 2016^23^

In 11 of the 20 countries studied (55%), urbanization was growing and overall mortality was decreasing as incomes rose. In all countries, noncommunicable diseases and associated mortality were both increasing. The proportion of deaths due to noncommunicable disease was greater in countries with higher incomes (Fig. 1). In 12 countries (60%), noncommunicable disease was a priority for primary health-care policy (Box 2), though countries were still addressing the substantial burden of communicable disease and, in some cases, violence and injury. There was a consensus that noncommunicable diseases should be tackled by increasing resources for preventive and promotive services. However, in some countries (e.g. Kenya), there was little financial support and payments for health promotion services were unreliable. Countries that prioritized community health workers (CHWs) and community-based service delivery generally reported better primary health-care services. In Ethiopia, for instance, health extension workers were regarded as critical for improving primary health-care coverage.

Box 2 **Noncommunicable disease medicine in a UHC scheme in Georgia, case studies of primary health-care systems in low- and middle-income countries, 2016–2018**

In Georgia, the cost of medicines for outpatients traditionally made up a high proportion of out-of-pocket health expenditure. In response, the government expanded the UHC benefits package in 2017 to cover noncommunicable disease drugs for four chronic conditions that accounted for more than 80% of the disease burden in the country, thereby including the prevention and management of noncommunicable diseases in primary health-care services.

UHC: universal health coverage.

### Governance and service models

In 70% of countries (14/20), primary health-care services were fragmented: they involved a wide range of public and private providers, nongovernmental organizations, faith-based organizations and traditional medicine providers (Box 3). Service delivery was affected by the perception that care quality at primary health-care facilities was low and by the lack of a gate-keeping mechanism in the health system. All countries had a basic, package-of-care approach to primary health care and, in 75% (15/20), CHWs and community-based services were building blocks of the health system. Yet, programmes for CHWs were weakened by under-resourcing, low pay, a dearth of supervision, poor definitions of roles, low motivation and morale, and poorly functioning referral processes, all of which hampered coordination and the continuity of care. In some countries (e.g. Bangladesh, Ghana and Uganda), decentralization of the health system had led to the fragmentation of primary health care, particularly where central governments retained control of the management of human resources for health, health financing and vertical programmes, and ring-fenced resources for certain programmes.

Box 3 **Health-care fragmentation in Bangladesh, Mexico and Nigeria, case studies of primary health-care systems in low- and middle-income countries, 2016–2018**

In Bangladesh, different government departments ran primary health-care services in urban and rural areas with little coordination between them, and the private sector was very strong in urban areas.

In Mexico, despite ongoing efforts to integrate the three main, primary health-care, public sector institutions using health-care service exchange agreements, fragmentation remained.

In Nigeria, the coordination of primary health care was limited by the presence of a wide array of providers, including faith-based organizations, nongovernmental organizations, the private sector, local government authorities, and national and state public institutions.

### Financing

**Fig. 2:**
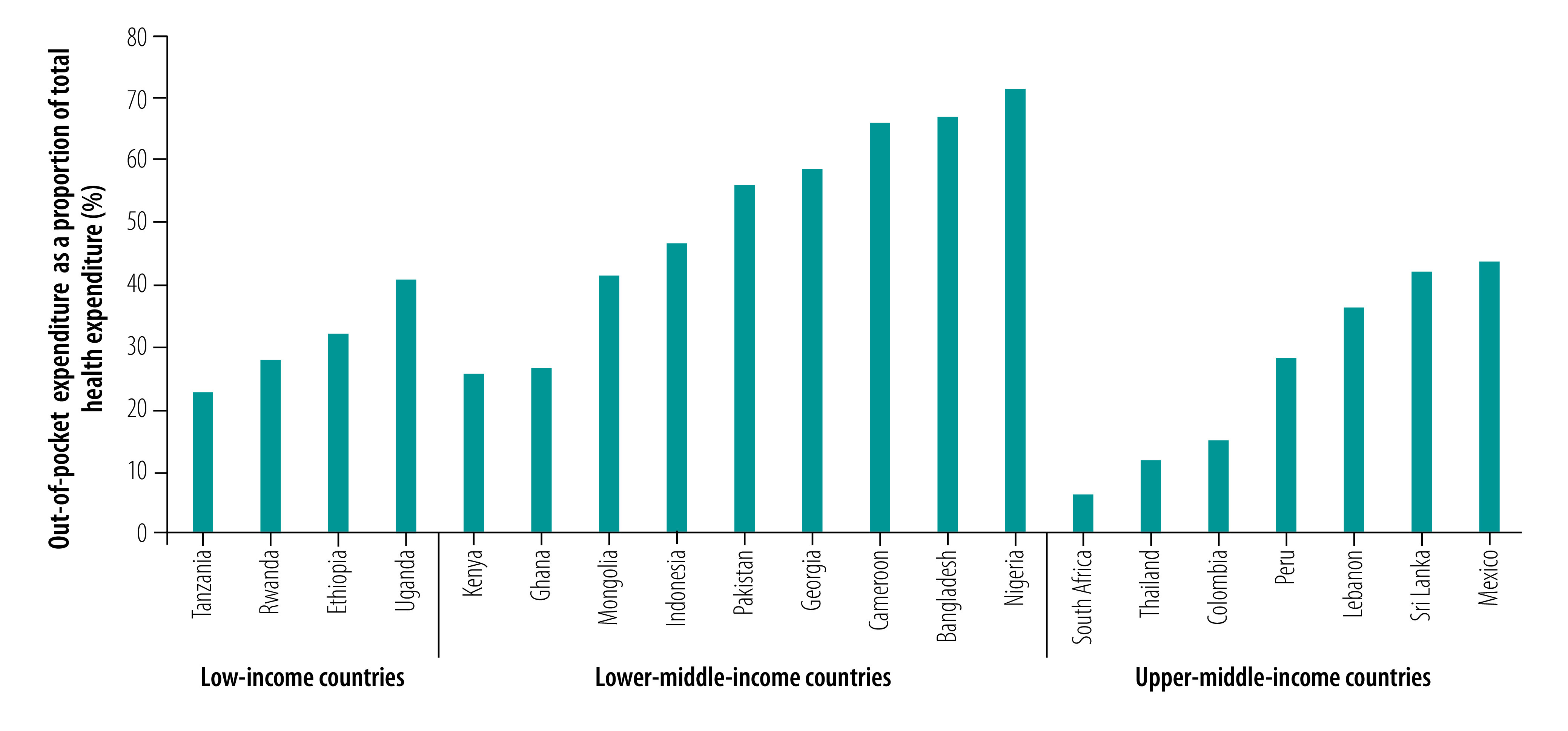
Out-of-pocket expenditure as a percentage of total expenditure on health, 20 low- and middle-income countries, 2014

Note: Case studies of primary health-care policy and systems were performed for all 20 countries under the Primary Health Care Systems (PRIMASYS) initiative.^15^

Commonalities in primary health-care financing across countries included: (i) policy commitments to reducing catastrophic health expenditure and improving equity; and (ii) movements towards financial protection and UHC and towards increasing primary health-care coverage using a risk-pooling mechanism. In 2014, out-of-pocket expenditure was over 40% of total health expenditure in 10 of the 20 countries (Fig. 2).^24^ In 65% (13/20), high out-of-pocket expenditure led to the development of a national health insurance or social health insurance scheme to expand UHC. The main challenges in developing such schemes included: integrating existing public and private systems; underfunding and corruption; scaling up insurance pilot projects; involving the private sector; and engaging workers in the informal sector.

Data source: World Health Organization.^24^

Current strategies to reduce out-of-pocket and catastrophic health expenditure are listed in Box 4. In Ethiopia, community-based health insurance schemes started in selected districts in four regions and were scaled up to include 205 districts (out of 558) nationally. In 2016, they covered 36% of the population in those districts. In Thailand, a universal coverage scheme introduced in 2002 substantially increased the government’s share of total health expenditure and reduced out-of-pocket expenditure.

Box 4 **Strategies for reducing out-of-pocket health expenditure, case studies of primary health-care systems in low- and middle-income countries, 2016–2018**

- abolish user fees and implement free maternal, newborn and child health services as a priority;
- fund services and infrastructure in poor, rural or remote areas;
- provide government-funded health insurance for poor people and for vulnerable population groups, such as refugees;
- level health insurance premiums in a progressive way;
- regulate health professionals’ service fees;
- ensure providers of contracted-out services (including nongovernmental organizations) provide services at prescribed rates; and
- establish a cash transfer system that targets certain services (e.g. maternal, newborn and child health) or poorer households and ensure government resources are distributed on an equitable basis (e.g. according to need or remoteness).

Overall, government funding as a percentage of gross domestic product was generally low and often health systems’ budgets were not aligned with indicators of workload or resource needs.

### Regulation and quality improvement

The licensing of primary health-care facilities presented a mixed picture. Good processes were reported in some countries, whereas systems were very weak or nonexistent in others (Box 5). In 70% (14/20), the private sector was largely unregulated and there were few incentives for regulatory compliance (e.g. strategic purchasing), which resulted in suboptimal qualification of health workers and poor quality of care. The lack of public oversight of heterogeneous groups of private providers and facilities can lead to the fragmentation of primary health-care, particularly in mixed systems where the private sector is dominant (e.g. in Bangladesh, Lebanon and Nigeria). However, in several countries, strategic purchasing was being introduced and there were innovative developments linking licensing with contracts, accreditation and insurance schemes (Box 6).

Box 5 **Regulatory challenges in Ghana and Kenya, case studies of primary health-care systems in low- and middle-income countries, 2016–2018**

In Ghana, different bodies were responsible for regulating health personnel, drugs and facilities, there was poor coordination between institutions, and monitoring and enforcement were difficult.

In Kenya, under-resourced regulators were not able to prevent facilities operating without licences or with unqualified staff.

Box 6 **Strategic purchasing in Indonesia and Lebanon, case studies of primary health-care systems in low- and middle-income countries, 2016–2018**

In Indonesia, only accredited facilities could receive payments from the national insurance body and primary health-care centres were accredited only if their staff had undergone special training in midwifery emergencies.

In Lebanon, contractual agreements between the health ministry and primary health-care facilities covered the supply of essential medicines. The contractual process was linked to the accreditation of primary health-care providers and dispensaries, which had to maintain care quality.

Fifteen countries (75%) had a national oversight body (similar to the United States Food and Drug Administration) responsible for regulating the manufacture, importation, registration, distribution and pricing of drugs and, sometimes, equipment. In 45% (9/20), the regulatory system was weak as there was limited capacity to monitor or enforce regulations, which led to problems with substandard and counterfeit drugs. Most countries (13/20) had issues with supply chain management that affected drug stocks and, in many countries, the regulation of traditional medicine was an emerging challenge. Improvements in the effectiveness of regulation have occurred where financial incentives were linked to regulation and quality improvement (e.g. in Indonesia and Lebanon) and where there was strong community involvement and oversight (e.g. in Ethiopia). 

In all countries, quality improvement was an area of weakness: little effort was being made to improve primary health-care performance and there were few incentives. In 70% (14/20), quality improvement structures and processes were in place but there were problems with benchmarking standards, funding for quality improvements and achieving gains in outcomes and performance.

### Policy-making

Frequently, primary health-care planning took place at the district level and was consolidated at regional and national levels – all countries had a national planning and budgeting process. In many cases, planning was a top-down process and there was little participation by low-level workers or communities. Consequently, primary health-care policy was not responsive to local needs or capacities. The main primary health-care policy issues were: adopting an integrated or sector-wide approach; decentralization; accountability for performance (Box 7); and social and financial risk protection schemes. In addition, there were often discrepancies between a primary health-care policy and its implementation in the real world.

Box 7 **Community engagement in policy-making in Ethiopia, case studies of primary health-care systems in low- and middle-income countries, 2016–2018**

In Ethiopia, health plans were embedded in five-year strategic development plans for growth and transformation. These plans were developed through extensive consultations that included communities and multiple stakeholders. Districts had autonomy, which allowed for both bottom-up and top-down planning processes. Districts could argue for resources during the budgetary process and district committees could develop health plans to suit the local context.

### Workforce

In 2017, the number of nurses and doctors combined ranged from 0.40 per 1000 population in the United Republic of Tanzania to 6.94 per 1000 in South Africa. In all 20 countries, the distribution of the health workforce, especially doctors and nurses, was uneven. In particular, rural areas were underserved: poor working conditions in rural areas was a disincentive in 35% of countries (7/20), whereas centralized decision-making and financial control were bottlenecks to recruitment and deployment in others. Absenteeism was common and was linked to poor working conditions, the absence of incentives and nonfunctioning regulatory and accountability mechanisms, particularly for doctors. Five countries (i.e. Bangladesh, Colombia, Georgia, Lebanon and Pakistan) had more doctors than nurses, whereas nurses predominated in all African countries studied. Professionals frequently worked simultaneously in both private and public sectors, which contributed to inequitable coverage. Traditional health practitioners were generally unregulated but Ghana, South Africa, Sri Lanka and Thailand were addressing regulation.

### Community participation and accountability

Two types of structure for community participation were identified: (i) elected representatives at national, regional, district and local government levels; and (ii) appointed community participation bodies that play an advisory role. Community bodies (e.g. district committees, community committees, hospital management boards and facility health committees) existed in 55% of countries (11/20) but wielded little power or influence. In countries where health services had largely been privatized (e.g. Georgia, Pakistan and urban Bangladesh), local government and community bodies had limited oversight of primary health-care services.

## Discussion

Our multicountry assessment found commonalities in the performance of primary health-care systems in low- and middle-income countries, derived lessons for improving performance in these countries and identified strategies for enhancing primary health-care policy-making and systems. Substantial improvements in primary health-care systems had been made in the 20 countries studied, including better community-based services. Overall, however, the effort and investment devoted to strengthening health promotion and prevention was not commensurate with the swiftly increasing morbidity and mortality associated with noncommunicable diseases. Frequently, a primary focus on curative services undermined the comprehensiveness and continuity of primary health care. 

Although primary health care was given a high priority in policy-making and agenda-setting in the countries studied, in practice it was limited by inadequate human and financial resources, by the limited availability and suboptimal quality of commodities, and by weak support systems. The predominant primary health-care delivery approach remained the provision of an essential package of health services and the principal focus was on high-impact, efficient and equity-enhancing interventions and services. Yet, there were weaknesses with the comprehensiveness, integration and quality of essential primary health-care interventions: for example, the inclusion of essential interventions in planned packages of primary health-care services was uneven and coverage was limited. Moreover, inequitable resource allocation and a lack of regulation and accountability also impeded the implementation and scaling up of high-quality primary health-care services. A UHC intervention compendium is being developed by WHO to inform the development and redesign of health benefits packages, while recognizing that the implementation of essential primary health-care services can vary across countries according to context, priorities, resources and capacities.^25^ Additional guidance is available on including essential services, such as comprehensive sexual and reproductive health and rights interventions,^26^ in health packages.^27^^,^^28^ Our study shows that additional attention and resources should be allocated to support essential primary health-care packages, including better governance, regulation and human resources and more funding.

Our analysis also highlights the importance of primary health-care systems being adaptive so that services can be scaled up and funding can be increased when more domestic resources become available. Moreover, the inclusion of essential primary health-care interventions in UHC benefits packages should be appropriate to the context, should be informed by evidence from local, disaggregated data and real-world knowledge, and should consider the views of primary health-care stakeholders (e.g. health-care providers, communities and the beneficiaries of services).^29^ Yet, our study found that many low- and middle-income countries lacked the resources, mechanisms and capacities needed to collect, appraise and use context-sensitive evidence on primary health care.

In addition, our study identified gaps in the measurement and reporting of primary health-care-sensitive indicators that can weaken the monitoring, development and accountability essential for improving primary health care. Our findings corroborate previous evidence that more sensitive metrics of primary health care, including financial indicators, are required and confirm the importance of strengthening health information and surveillance systems.^30^ Our study also identified the need for increased capacity to use evidence to strengthen primary health-care systems and for more incentives to use that evidence. Local data and research are essential for assessing the implementation of primary health care and for establishing the effectiveness of any innovations introduced or any activities scaled up within the existing health system, for instance digital primary health-care interventions.^29^^,^^31^ Furthermore, our analysis found gaps in context-sensitive knowledge about the most effective ways of: (i) enabling primary health-care systems to adapt to local needs and to the needs of the population in general; and (ii) enhancing social accountability and the community engagement and empowerment pillar of primary health care.

Support for CHWs could be strengthened by providing adequate resources and effective referral systems, by ensuring proper recognition, training and supervision, and by enforcing regulations. Although guidance is now available on how health policy and systems can help improve community health worker programmes,^32^ sustainable funding for workers and community services is also required.^33^ Funding and support for quality improvement schemes are essential, particularly when task-shifting to CHWs, nurses and midwives is taking place for some primary health-care services, such as maternal or mental health care.^34^

Regulation and accountability are important for mixed health systems that involve fragmented, heterogeneous, private-sector provision, such as systems where the private sector is dominant (e.g. in Bangladesh, Lebanon and Nigeria), systems with private-sector hospitals and clinics for better-off people and systems with extensive use of private drug shops (e.g. in Ghana, Nepal and the United Republic of Tanzania).^35^ There are innovative ways of enhancing care coordination and of improving the continuity of care across health-care levels (e.g. primary to secondary care) in mixed-market health systems, such as intermediary organizations that enable government to engage with numerous private sector actors while supporting accountability and quality improvement.^36^

Maintaining inclusive, universal mechanisms that protect against high out-of-pocket spending remains a challenge, for example, in countries with good service coverage but a high level of financial hardship.^37^ As catastrophic health expenditure and poverty due to health-care expenditure are still increasing globally,^37^ equity-enhancing financing schemes are needed to enable primary health-care systems to respond to the evolving needs of vulnerable population groups. Our study found that public financing of primary health care needs to be strengthened and sustained. Innovative primary health-care financing schemes are emerging, such as blended finance, where strategic development funding is used to mobilize private capital flows (e.g. for immunization).^38^ Transparency and accountability in financial management can be improved by strengthening public financial management systems, which can also help improve the efficiency of primary health-care spending.^39^ In 2019, WHO called on governments to redouble effort to expand primary health-care coverage and to spend at least 1% more of their gross domestic product on primary health care.^37^

Table 2 summarizes the key lessons learnt from our study and lists options for strengthening primary health-care systems and financing derived from the evidence and from guidance on policy and systems decision-making.

**Table 2:** Challenges faced by, and options for strengthening, primary health-care systems, case studies of 20 low- and middle-income countries, 2016–2018

Aspect of primary health-care system	Issues identified in primary health-care systems in PRIMASYS countries^a^	Options for strengthening primary health-care systems
Primary health-care services	• Mortality and morbidity due to noncommunicable diseases increasing; • fragmented and inequitable service delivery and coverage linked to a multiplicity of primary health-care providers and stakeholders; • suboptimal community health services and outreach; and • quality improvement schemes generally weak, albeit that quality improvement is increasingly recognized as a priority for primary health-care performance	• Enhance preventive services across the system and improve their financing and resource allocation;^29^ • improve regulation and accountability of primary health-care providers, particularly in the private sector;^40^ • embed equity indicators in primary health-care monitoring and accountability schemes;^29^ • improve support systems for community-based service delivery, including digital interventions;^29^^,^^32^ and • integrate effective and context-sensitive quality improvement schemes into primary health-care strengthening initiatives^29^
Financing	• In most countries, national or social health insurance schemes are considered a mechanism for reducing out-of-pocket and catastrophic health expenditure and for moving towards UHC; and • primary health care remains underfunded by public financing schemes	• Incorporate equity-enhancing financing schemes into primary health-care strengthening efforts;^29^ • address barriers to the implementation of pro-poor primary health-care policies;^31^ • sustain and strengthen public financing of primary health-care systems;^29^^,^^37^ • ensure governments commit to increase spending on primary health care by at least 1% of GDP;^37^ and • enhance transparency and accountability in financial management systems^39^
Governance and regulation	• Poor integration of primary health care associated with multiple insurance schemes, vertical programmes and role conflicts between levels of care; • decentralization initiatives leading to fragmentation of primary health care when resources are not distributed adequately; and • difficulties with regulating the private sector and addressing substandard and counterfeit drugs	• Develop a coherent primary health-care organizational framework with well delineated roles and responsibilities;^29^ • ensure decentralization schemes pay greater attention to primary health-care resource allocation (including budgets) and improve their accountability mechanisms;^41^ • engage primary health-care stakeholders in decentralization initiatives to enhance the responsiveness and people-centred nature of health systems;^42^ and • strengthen regulatory mechanisms for: (i) accreditation and monitoring of training institutions; (ii) licensing and regulation of facilities; (iii) quality oversight of the medicines supply chain; and (iv) dealing with regulatory infractions^40^
Policy-making and implementation	• People-centred nature and responsiveness of primary health-care policies undermined by a lack of participatory approaches to planning and implementation; and • valid data and contextualized evidence underused in primary health-care policy planning and implementation and in scaling up services	• Support co-development approaches to primary health-care policy-making and implementation, with a strong focus on community participation;^29^^,^^31^ • integrate primary health-care strategies into intersectoral action, social protection and policies addressing the social determinants of health;^29^ and • strengthen primary health-care surveillance systems, including the use of primary health-care-sensitive data within health management information systems, and strengthen the capacity to collect and use local primary health-care data^30^^,^^37^
Workforce	• Task-shifting from specialist physicians to general practitioners and nurses and from mid-level health cadres to community health workers and community health volunteers; • distribution of health-care workers biased towards urban areas; • simultaneous contributions from public and private sectors linked to inequitable coverage; and • qualification standards of health workers not maintained	• Improve training and support systems for task-shifting and pay greater attention to incentives, career paths and enforcement by regulatory bodies;^29^^,^^34^ • align licensing and regulation with accreditation and insurance schemes to create functional checks and balances;^40^ • ensure that mechanisms to promote adequate primary health-care staffing in rural areas address working conditions and provide context-appropriate incentives linked to human resources for health, satisfaction and motivation;^43^ • enhance the licensing, certification and regulation of health-care workers, including private primary health-care providers and traditional health practitioners;^40^ and • link relicensing to a strong, continuous, professional development system^40^
Community engagement and empowerment	• Little consideration given to community and social accountability structures; and • low level of community engagement by privatized primary health-care services	• Integrate community and social accountability mechanisms into primary health-care strengthening initiatives (e.g. embed community needs into accreditation processes);^29^^,^^42^ and • establish formalized structures and processes for community participation (e.g. through legislation)^42^

GDP: gross domestic product; PRIMASYS: Primary Health Care Systems; UHC: universal health coverage.

^a^ The 20 PRIMASYS (Primary Health Care Systems) countries were Bangladesh, Cameroon, Colombia, Ethiopia, Georgia, Ghana, Indonesia, Kenya, Lebanon, Mexico, Mongolia, Nigeria, Pakistan, Peru, Rwanda, South Africa, Sri Lanka, Thailand, Uganda and the United Republic of Tanzania.

Applying a complex, adaptive, health-systems approach to primary health care can also illuminate the interdependency of primary health-care domains and the system-level effects of primary health-care interventions.^44^ In Georgia, for instance, the introduction of a national health insurance scheme resulted in personnel moving to the private sector and to the secondary care level because the scheme entitled both public and private providers to bid for contracts and did not incentivize primary health care. This approach created a negative feedback loop between financing and the primary health-care workforce.

Our study’s findings were limited by the skewed distribution of the PRIMASYS countries towards middle-income countries. Moreover, although the case studies addressed policy- and systems-level determinants of primary health care, the nature and complexity of the evidence made it difficult to associate the performance of a primary health-care system or an improvement in primary health-care policy or implementation with a specific factor or initiative. In addition, countries used different definitions of primary health care: for example, whether or not district hospitals are included in primary health care. The analysis was also limited by the absence, under-reporting and suboptimal quality of data on primary health-care performance. Finally, we acknowledge that primary health-care systems and policies might have evolved since we collected the data.

## Conclusion

We have identified several priorities to improve primary health-care systems in low- and middle-income countries. In particular, communities should be engaged in a meaningful manner and primary health care should better adapt to evolving population needs. These actions will improve the people-centred nature and responsiveness of health systems. As public resources increase, primary health-care systems are well positioned to support the development of schemes that provide essential packages of health services and UHC benefits. In fact, strengthening primary health-care interventions and existing models for providing essential packages of health services are critical for achieving UHC and other health-related sustainable development goals between 2020 and 2030. Greater transparency and public accountability are also vital for setting priorities for essential benefits packages and primary health-care policy-making and for ensuring equity and human rights. For example, some low- and middle-income countries have embedded a rights-based approach to strengthening primary health-care systems by including sexual and reproductive health and rights interventions in primary health-care and UHC policies.

The COVID-19 pandemic demonstrates that strong primary health care is the cornerstone of preparedness and response planning, particularly for infection prevention and control. In addition, primary health care can act as a strategic entry point for multisectoral initiatives and for addressing issues around the political, commercial and legal determinants of health. For instance, the rapid rise in noncommunicable disease is expected to impede poverty reduction initiatives in low-income countries because household expenditure on health care will increase. By promoting the integration of health and social services, multisectoral collaboration, continuity of care and equity-enhancing schemes, primary health care can help address these complex challenges and accelerate progress towards UHC and the sustainable development goals.
